# The feasibility and efficacy of pedicle fixation by the Wiltse approach in the thoracic spine

**DOI:** 10.3389/fsurg.2024.1406111

**Published:** 2024-06-20

**Authors:** Lu Hao, Yufeng Xiang, Junhui Liu

**Affiliations:** ^1^ Department of Radiology, Sir Run Run Shaw Hospital, School of Medicine, Zhejiang University, Hangzhou, China; ^2^ Department of Orthopaedic, Linhai Second People's Hospital, Taizhou, China; ^3^ Department of Orthopaedics, Sir Run Run Shaw Hospital, School of Medicine, Zhejiang University, Hangzhou, China

**Keywords:** Wiltse approach, conventional transmuscular approach, pedicle fixation, thoracic diseases, multifidus muscle

## Abstract

**Study design:**

Retrospective Cohort Study.

**Objectives:**

To explore the feasibility and assess the efficacy of pedicle fixation with the Wiltse approach in the thoracic spine.

**Summary of background data:**

The current application of Wiltse approach is mainly practiced in the lumbar and thoracolumbar spines. Its application in the thoracic spine, however, has received little attention, especially in cases that requires only pedicel screw fixation without spinal decompression.

**Methods:**

The study analyzed the clinical records of consecutive patients with thoracic diseases who underwent pedicle fixation with either Wiltse or the conventional transmuscular approach (Wiltse group: 60 cases; Transmuscular group: 48 cases). Perioperative parameters, Visual Analogue Scale (VAS) scores, accuracy of pedicle screw placement, dead space between the muscles, Magnetic Resonance Imaging (MRI) appearance, electrophysiological changes in the multifidus muscle were compared between the two groups.

**Results:**

Compared with the transmuscular group, the Wiltse group was significantly better in blood loss and postoperative VAS scores. No difference was observed in incision length, operation time, and hospital stay. The dead space between the muscle cross-sectional region in the transmuscular group was 315 ± 53 mm^2^, and no dead space was found in the Wiltse group. On MRI images, the multifidus cross-sectional area (CSA) in the Wiltse group between the preoperative period and the last follow-up reduced by only 10.1%, while transmuscular group showed 46.1% CSA reduction. Electrophysiologically, the median frequency slope of the transmuscular group grew by 47.8% with average amplitude reduced by 16.4% between the preoperative period and 12-month postoperative.

**Conclusion:**

The Wiltse approach for pedicle fixation in the thoracic spine is a feasible and effective treatment, with fewer traumas and reliable clinical results. In particular, the Wiltse approach reduces postoperative dead space between the muscles and causes less atrophy in the multifidus muscle.

## Introduction

With respect to the choice of operative approach for thoracic diseases, the conventional transmuscular approach for posterior pedicle screw instrumentation is extensively used. However, the long incisions, wide muscle detachment from the spinal processes, and prolonged retraction can lead to ischemic necrosis and paraspinal musculature denervation (1, 2). Besides, paraspinal muscle injury is inevitable in posterior spinal operation. Therefore, it is necessary to formulate an optimized surgical method or approach to reduce the risk of iatrogenic muscle injury and better meet the surgical safety needs.

Due to scar formation and denervation after the conventional transmuscular approach for posterior pedicle screw instrumentation, the function of paravertebral muscles will change significantly (1). Relevant studies found that after the surgery, the strength of trunk muscles will decrease (3, 4). Spinal instability and severe back pain will also occur (1, 5, 6). To decrease the risk of iatrogenic muscle injury and method-associated morbidity, Wiltse (7) proposed a paraspinal sacrospinal fissure approach to lumbar spine in 1968 to avoid complications mentioned above. At present, this surgical approach is widely used. Compared with the traditional surgery, the Wiltse approach is superior in terms of intramuscular pressure (8), blood loss (9–12), serum creatine kinase (13) and back pain (11, 12, 14). However, the current application of this approach is mainly practiced in the lumbar and thoracolumbar spines. Its application in the thoracic spine has received little attention, especially in cases that requires only pedicel screw fixation without spinal decompression.

Accordingly, we analyzed the clinical records of consecutive patients with thoracic diseases, including suppurative spondylitis, spinal tuberculosis, and thoracic fractures, who underwent pedicle fixation (PF) with either Wiltse or the conventional transmuscular approach. The two groups were compared for perioperative parameters, dead space between the muscles, VAS scores, MRI appearance, and electrophysiological variations in the multifidus muscle. The study aimed to explore the feasibility and assess the efficacy of PF using the Wiltse approach in thoracic spine, thereby providing evidence for selecting surgical approach in the thoracic spine.

## Materials and methods

### Inclusion criteria

(1) Patients with thoracic diseases (suppurative spondylitis or spinal tuberculosis) who underwent anterior debridement and posterior pedicle screw fixation only, with no need for posterior spinal canal decompression. (2) Patients with fresh thoracic vertebral fracture who received posterior pedicle screw fixation only, with no need for posterior spinal canal decompression. (3) Patients with old thoracic vertebral fracture who received posterior pedicle screw fixation only, with no need for posterior spinal canal decompression or posterior spinal osteotomy. (4) Follow-up period ≥1 year.

### Exclusion criteria

(1) Patients with thoracic diseases (suppurative spondylitis or spinal tuberculosis) who needed posterior spinal canal decompression. (2) Patients with fresh thoracic vertebral fractures accompanied with dislocation that required paraspinal muscle stripping for reduction, or required posterior spinal canal decompression. (3) Patients with old thoracic vertebral fractures requiring posterior spinal canal decompression or posterior spinal osteotomy. (4) Non-compliant patient with no follow-up.

### Baseline clinical data

The study analyzed the clinical records of consecutive patients with thoracic diseases, including suppurative spondylitis, spinal tuberculosis, and thoracic fractures, who underwent pedicle screw fixation using either Wiltse or transmuscular approach. Patients were divided into two groups on basis of the last digit of their patient numbers, which was either even or odd. Up to 108 cases were included from February 2011 and December 2020 (Wiltse group: 60 cases; Transmuscular group: 48 cases). In the Wiltse group, 24 patients had suppurative spondylitis, 20 had spinal tuberculosis, and 16 had thoracic vertebra fractures. In the transmuscular group, 20 patients had suppurative spondylitis, 18 had spinal tuberculosis, and 10 had thoracic vertebra fractures. No significant differences exist between the two groups regarding gender, age, disease species, number of pedicle screws, or follow-up period (*P* > 0.05, Table 1). Ethical approval was obtained from the Ethics Committee of our hospital. Each patient has given their informed consent.

**Table 1:** Patients’ data are compared between the two groups.

	Wiltse approach	Transmuscular approach	*P* value
Patient number	60	48	
Gender (M/F)	32/28	26/22	0.817^a^
Age (mean ± STD)	63.5 ± 5.8	66.2 ± 6.1	0.069^b^
Disease species			0.845^a^
Suppurative spondylitis	24	20	
Spinal tuberculosis	20	18	
Thoracic vertebra fractures	16	10	
Number of pedicle screws	9.2 ± 2.9	9.8 ± 2.6	0.720^b^
Follow-up period (month)	14.7 ± 1.9	15.1 ± 3.4	0.816^b^

^a^ Chi-square test for proportions.

^b^ *t*-test for the differences between means.

### Surgical procedures

#### The Wiltse approach

**Figure 1:**
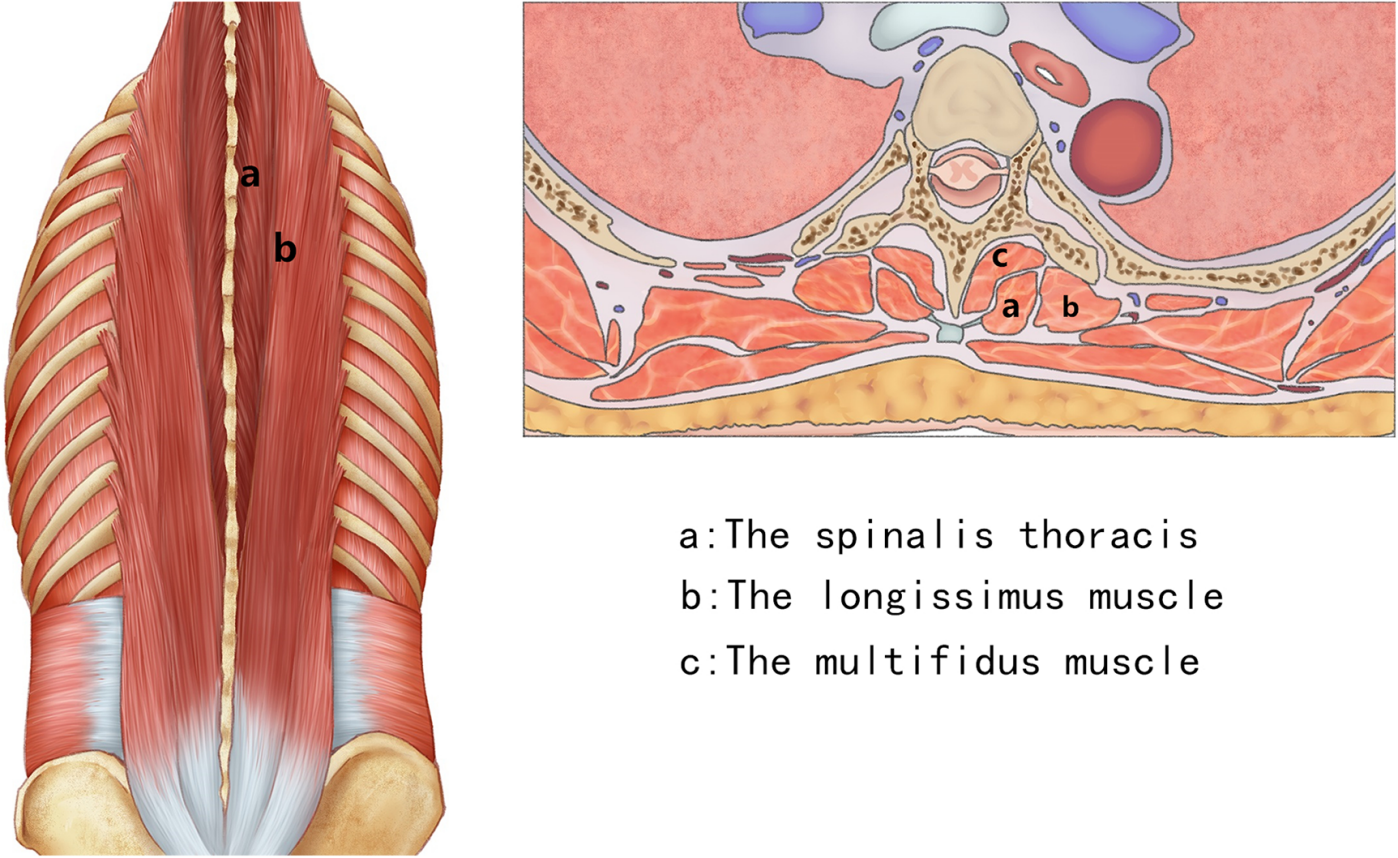
The Wiltse approach was approached by the interspace between the spinalis thoracis and longissimus muscles.

**Figure 2:**
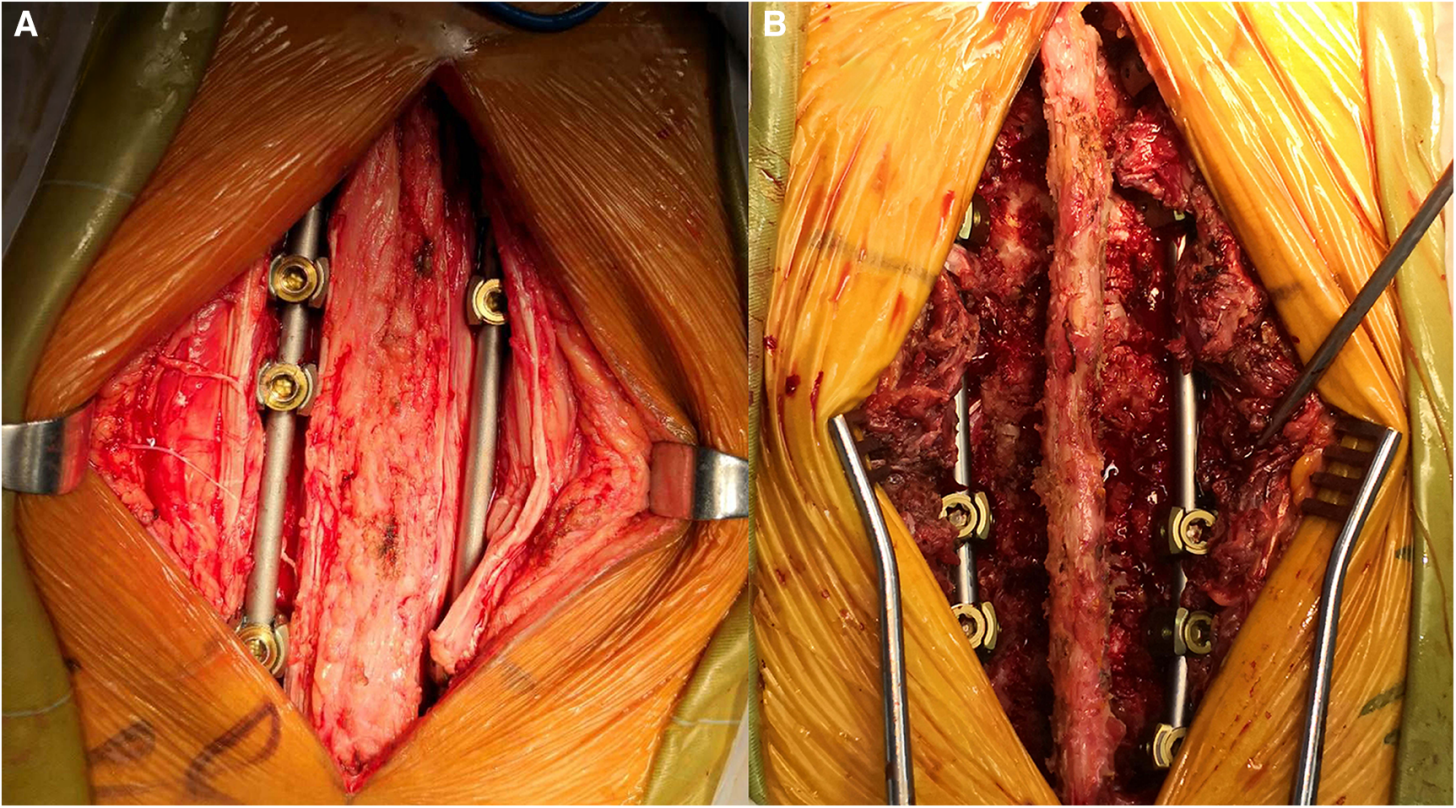
Pedicle fixation by the Wiltse (**A**) and transmuscular approach (**B**).

The patient was placed in a prone position under general anesthesia. The skin was then prepared with povidone iodine and draped in a sterile manner. A posterior midline incision was conducted in designed segments. The incision length depended on the number of levels to be explored, and the deep fascia were opened. For T1–T4 segments, the trapezius and rhomboid aponeurosis should be cut open; for T4–T6 segments, only the trapezius aponeurosis needed to be cut open; for T6–T12 segments, the trapezius aponeurosis and latissimus dorsi aponeurosis needed to be cut open, then processed by the interspace between the spinalis thoracis and longissimus muscle (Figures 1, 2). After appropriate stripping, the outer edges of the articular were exposed, and the pedicle screw entry point was determined (The entry point was chosen to be the intersection between the midlines of the facet joint and transverse process).

#### The conventional transmuscular approach

For the traditional transmuscular approach, body position placement, anesthesia, and sterilization were conducted as the same in the Wiltse approach. During the operation, the posterior midline incision was made at the targeted segments, and the paraspinal muscle was stripped. The pedicle screw entry points were then determined the same way as the Wiltse approach.

None of the patients in our study underwent posterolateral arthrodesis. All operations were made by the same team. Perioperative parameters (incision length, blood loss, surgery duration and hospital stay) were recorded. The VAS scores were evaluated preoperatively, after 1 week, and at the last follow-up. Serum creatine kinase levels (a measure of muscle damage) were assessed preoperatively, and 1, 3, and 5 days after the surgery.

#### MRI evaluation

MRI was performed preoperatively (internal fixation) and at the last follow-up to assess the fatty infiltration level. All internal fixation artifacts applied in operation were MRI compatible. The transverse section images for every disc superior and inferior to the target vertebra were chosen through the location of lines on sagittal plane scans of MRI. Based on the digital image processing software, the collected image is analyzed to determine the cross-sectional region of the multifidus muscle. The ROI area was established around the muscle of the target area on both sides of the spine, and the interference of nearby bone structure and soft tissue was avoided. The criteria established by Goutallier et al. were referred to when grading the severity of fat infiltration in the multifidus muscle: Grade A, normal muscle; Grade B, fat tissue sparsely distributed between the muscle fibers; Grade C, fat tissue almost equal to the muscle fibers; and Grade D, more fat tissue than muscle fibers (15).

#### CT evaluation

CT was performed 1 day after the operation to evaluate the pedicle screw placement precision and the dead space between the muscles. The CSA of the dead space between the muscles was determined based on digital image software (Image J, Bethesda, MD, USA).

#### Electromyography (EMG)

The recording of EMG was made before the surgery and 12 months after the surgery. During the examination, the patient lied prone on the examination table and contract the back muscles, and then carry out the Billing Sorenson test (16). Matching electrodes and Mega-T8 sEMG monitor were used to record the EMG for 30 s. All the subjects were tested three times, with a rest of 3 min, which can reduce the interference between different tests and improve the accuracy of the results. The median frequency slope (MFs) and mean EMG amplitude values and other indicators were calculated, and then compared between groups and within groups.

#### Data analysis

Data were input into Excel (Microsoft, Redmond, WA, USA) and quantitative results were expressed in terms of mean and standard deviation. Data were transferred to the SPSS 20.0 software (PASW, Statistics, IBM, USA). Group t-tests (two-sided) and chi-square tests were used to compare mean values and proportions, respectively. A significance level of 0.05 was used, and *P* < 0.05 was considered significant without multiple test adjustment.

## Results

### Perioperative parameters

Compared with the transmuscular approach, the Wiltse approach was significantly advantageous in terms of blood loss (*P* < 0.05). No significant differences were found in length of hospital stay, operation time and length of incision (*P* > 0.05) (Table 2).

**Table 2:** Comparison of perioperative parameters between the two groups.

	Wiltse approach	Transmuscular approach	*P* value
Patient numbers	60	48	
Length of incision (mm)	144.3 ± 10.6	146.4 ± 12.4	0.322
Operation time (min)	114.5 ± 9.6	104.8 ± 11.7	0.083
Blood loss (ml)	70.2 ± 5.9	128.3 ± 9.1	0.042
Hospital stay (days)	14.1 ± 1.6	15.2 ± 1.3	0.143

Values represent the mean ± standard deviation.

### Serum creatine kinase level

**Figure 3:**
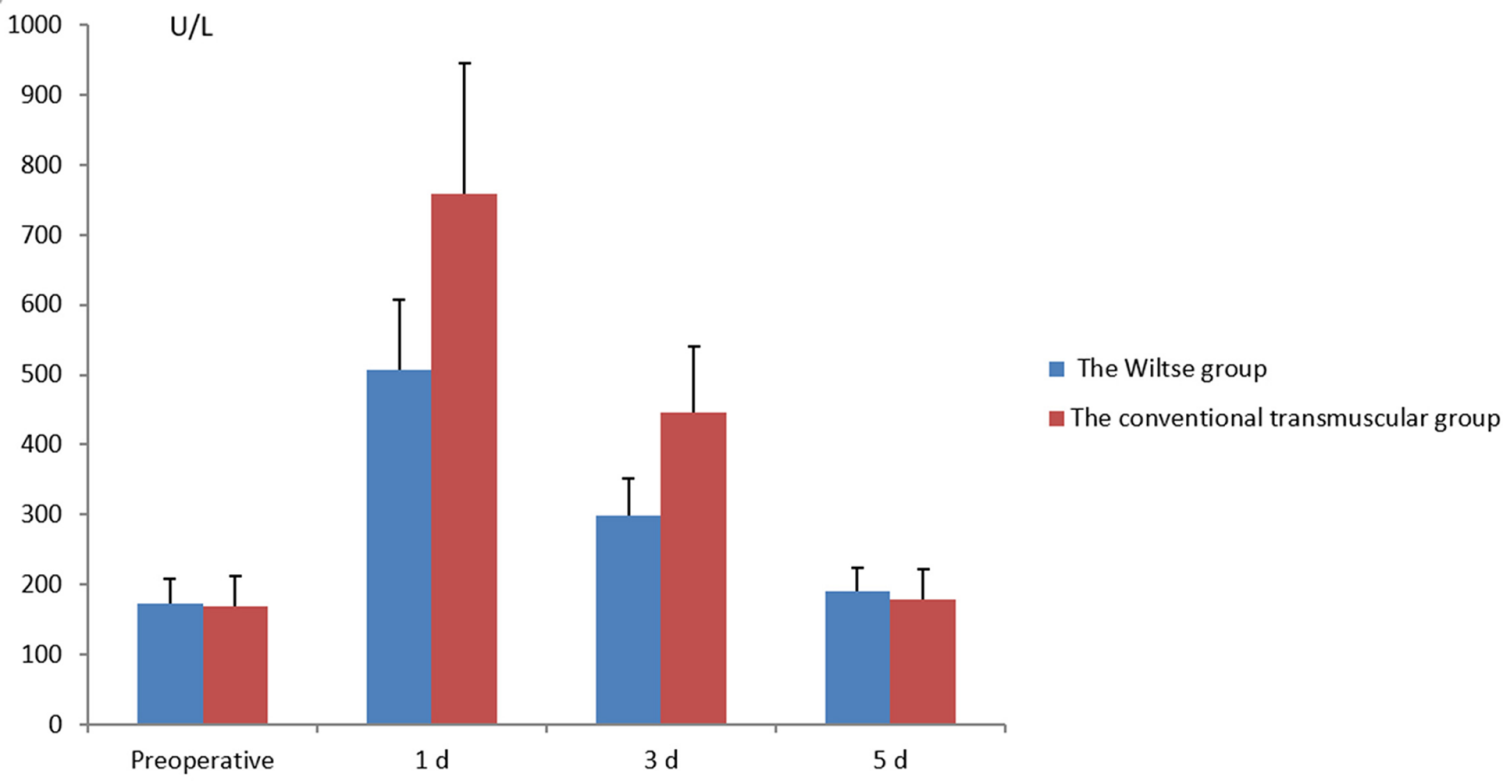
Comparison of pre- and postoperative serum creatine kinase levels between the different groups.

No statistical difference was found in serum creatine kinase level between the two groups before operation (*P* > 0.05). The transmuscular group showed significantly higher degree of serum creatine kinase level compared with the Wiltse group on the 1st and 3rd day after surgery (*P* < 0.05), but the difference was not significant on the 5th day. (*P* > 0.05) (Figure 3).

### VAS scores

As shown in Table 3, the preoperative VAS scores showed no significant differences between the two groups (*P* > 0.05). The postoperative VAS scores improved compared with the preoperative VAS scores in both groups (*P* < 0.05). The VAS improvement at the last follow-up was higher in the Wiltse group than that in transmuscular group (*P* < 0.05).

**Table 3:** Comparison of preoperative and postoperative VAS scores between the two groups.

	Wiltse approach	Transmuscular approach	*P* value
Preop VAS	7.3 ± 1.4	7.2 ± 1.7	0.286
Postop (day 7) VAS	1.5 ± 0.2	3.9 ± 1.1	0.037
Last follow-up VAS	0.9 ± 0.2	1.9 ± 0.3	0.046

Values represent the mean ± standard deviation.

### CT evaluation

**Figure 4:**
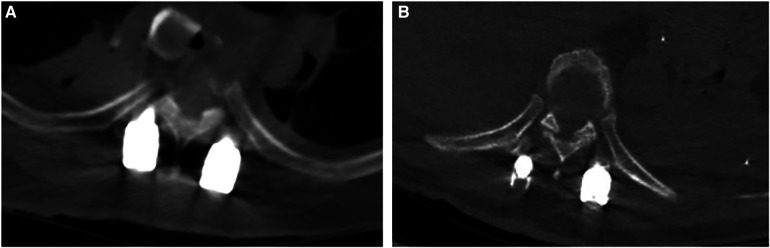
The dead space between the muscle can be seen in the transmuscular group (**A**), and no dead space was found in the wiltse group (**B**).

The dead space between the muscle CSA in the transmuscular group was 315 ± 53 mm^2^, and no dead space was found in the Wiltse group. No screw was misplaced outside the pedicle in either group (Figure 4).

### MRI evaluation of the multifidus muscle

**Figure 5:**
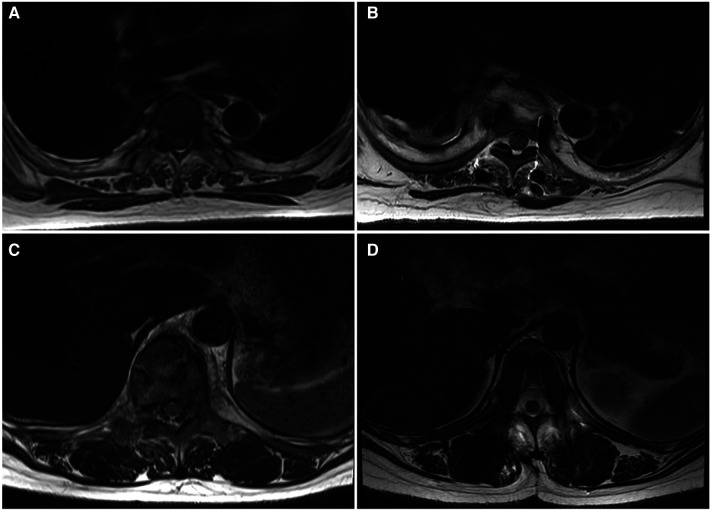
MRI axial view of the lumbar spine and muscles; the Wiltse group [(**A**) preop, (**B**) last follow-up] shows less multifidus atrophy compared to the transmuscular group [(**C**) preoperative, (**D**) last follow-up].

For the Wiltse group, multifidus CSA reduced by only 10.1% (*P* > 0.05) between the preoperative period and last follow-up (Figure 5). In the transmuscular group, the multifidus CSA reduced by 46.1% (*P* < 0.05) at the same time point. The multifidus CSA at final follow-up was significantly less than that in the Wiltse group (*P* < 0.05) (Table 4). The grade of fatty infiltration in multifidus muscle was bilaterally assessed. For the Wiltse group, fatty infiltration was grade A in 22 cases, B in 30 cases, and C in 8 cases preoperatively. Postoperative evaluation showed grade B in 41 cases, C in 16 cases, D in 3 cases at final follow-up (Table 5). In the transmuscular group, fatty infiltration was grade A in 18 cases, B in 24 cases, C in 6 cases preoperatively. Postoperative evaluation showed grade B in 8 cases, C in 26 cases, D in 14 cases at final follow-up (Table 5).

**Table 4:** Comparison of the pre- and postoperative cross-sectional area (CSA) of the multifidus muscle in the two groups.

	Wiltse approach	Transmuscular approach	*P* value
Patients (*n*)	60	48	
Preop (mm^2^)	192.3 ± 20.7	187.8 ± 18.3	0.384
Last follow-up (mm^2^)	172.8 ± 22.8	101.3 ± 10.3	**0.029**

Values represent the mean ± standard deviation.

**Table 5:** Fatty infiltration grade (number of cases) in the two groups.

	Wiltse approach				Transmuscular approach			
	A	B	C	D	A	B	C	D
Preoperative	22	30	8	0	18	24	6	0
Last follow-up	0	41	16	3	0	8	26	14

### EMG

In the transmuscular group, MFs was significantly enhanced by 47.8% (*P* < 0.05), with Average amplitude (AA) significantly reduced by 16.4% (*P* < 0.05) between the preoperative period and 12 months postoperatively (Table 6). There was no significant difference (*P* > 0.05) in the Wiltse group. At 12 months postop, AA was significantly lower and MFs was significantly larger in the transmuscular group compared with that of Wiltse group (*P* < 0.05).

**Table 6:** EMG slope (MFs) and average amplitude (AA) values in the two groups, both preop and 12 months postop.

	Wiltse approach	Transmuscular approach	*P* value
Preop MFs (Hz/min)	3.14 ± 1.43	3.09 ± 1.24	0.201
Postop (1 year) MFs (Hz/min)	3.89 ± 1.21	5.93 ± 1.17	0.048
Preop AA (μV)	78.4 ± 5.2	79.1 ± 3.3	0.191
Postop (1 year) AA (μV)	74.2 ± 5.6	66.1 ± 4.6	0.037

The *P*-value represents the comparison of Transmuscular vs. Wiltse approach at selected time points.

Values represent the mean ± standard deviation.

## Discussion

### The feasibility of pedicle fixation using the Wiltse approach in the thoracic spine

Anatomy shows that the paraspinal muscles from T1–T4 and from T6–T12 are divided into superficial, middle, and deep layers. The superficial and deep layers have the muscle components that stayed constant for both segments where the trapezius forms the superficial layer, and the multifidus, spinalis thoracis, and longissimus form the deep layer. The middle layer has a different layout, where rhomboideus covers T1–T4 and latissimus dorsi covers T6–T12. The muscle layout from T4–T6 is different from other segments of the thoracic spine. There are only two layers from T4–T6 in which trapezius forms the superficial layer and multifidus, spinalis thoracis, and longissimus in deep. Therefore, in the Wiltse approach, for T1–T4 segments, the trapezius and rhomboideus aponeurosis should be cut open; for T4–T6 segments, only the trapezius aponeurosis needed to be cut open; and for T6–T12 segments, the trapezius aponeurosis and latissimus dorsi aponeurosis needed to be cut open, then processed by the interspace between the spinalis thoracis and longissimus muscles. After appropriate stripping, the outer edges of the articular and transverse process can be exposed, and the pedicle screw entry point can be determined. The interspace can be easily found in the lower thoracic vertebrae; therefore, in the lower thoracic vertebrae, we first seek out the interspace in the caudal regions, and then find all the interspaces toward the cephalad regions.

### Advantages of PF by the Wiltse approach in the thoracic spine

Pedicle screw instrumentation has been extensively adopted in the management of thoracic diseases (suppurative spondylitis, spinal tuberculosis, or thoracic fractures). Using traditional operation approach, the multifidus muscle is most easily affected, and the serum creatine kinase level will be significantly increased after muscle injury. Therefore, serum creatine kinase level was routinely selected in the evaluation of early postoperative muscle injury (13, 17, 18). A study found that the activity of serum creatine kinase increased after operation, reached the peak on the first day, and then decreased continuously, and basically returned to normal on the fifth day (17). Similar to the results reported by Kim et al. (13), our outcomes showed that the Wiltse group displayed lower the serum creatine kinase level than the transmuscular group on the first and third days after operation, and the results were statistically different.

When the consequences of muscle injury were evaluated on MRI, the reduction of muscle CSA and the deposition of connective tissue were the main factors, which can be reflected by the signal intensity change of the late T2-weighted image (3). Previous research has shown muscle swelling due to edema up to 10 months postoperatively (3, 19). In the current study, the percent decline in the lean multifidus muscle CSA was significantly lower in the Wiltse group than that in the transmuscular group. Integrated with the outcomes in the literature (8, 13), our findings confirm that the Wiltse approach brings less muscle injury than the traditional transmuscular approach.

The paraspinal muscle atrophy can be evaluative using comparative analysis since EMG signal conduction velocity will decrease in atrophic muscles (20, 21). This reduction can be detected in EMG analysis based on the reduction of AA value and the higher MFs (22). Compared with the Wiltse group, the transmuscular group showed lower AA and higher MFs, showing more severe muscle atrophy in the transmuscular group (20, 21).

Moreover, the Wiltse approach for pedicle fixation reduces postoperative dead space between the muscles, which can decrease the risk factors of postoperative infection and the tension of suture, while avoiding postoperative infection.

There are several limitations in our research. This was a single-center, cross-sectional research with a small sample size and short follow-up duration. It focused on the multifidus, and no related mechanisms were researched. In addition, significant metallic artifacts may bias our outcomes on CSA and fat infiltration evaluation. Further research is required in the future.

## Conclusion

The Wiltse approach for PF in the thoracic spine, which preserves the posterior ligament complex, is feasible and is an effective treatment, with fewer traumas, less bleeding, and reliable clinical results. In particular, the Wiltse approach reduces postoperative dead space between the muscles, which can decrease the risk factors of postoperative infection and the tension of suture. The Wiltse approach also causes less atrophy in the multifidus and reduces postoperative low back pain. It is an easy-to-learn method that worth to be suggested in the thoracic spine for pedicle fixation.

## Data Availability

The original contributions presented in the study are included in the article/Supplementary Material, further inquiries can be directed to the corresponding author.
